# Vision-Based Flying Obstacle Detection for Avoiding Midair Collisions: A Systematic Review

**DOI:** 10.3390/jimaging9100194

**Published:** 2023-09-25

**Authors:** Daniel Vera-Yanez, António Pereira, Nuno Rodrigues, José Pascual Molina, Arturo S. García, Antonio Fernández-Caballero

**Affiliations:** 1 Albacete Research Institute of Informatics, Universidad de Castilla-La Mancha, 02071 Albacete, Spain; 2 Computer Science and Communications Research Centre, School of Technology and Management, Polytechnic Institute of Leiria, 2411-901 Leiria, Portugal; 3 Institute of New Technologies—Leiria Office, INOV INESC INOVAÇÃO, Morro do Lena—Alto do Vieiro, 2411-901 Leiria, Portugal; 4 Departamento de Sistemas Informáticos, Universidad de Castilla-La Mancha, 02071 Albacete, Spain

**Keywords:** midair collision, obstacle detection, computer vision, systematic review

## Abstract

This paper presents a systematic review of articles on computer-vision-based flying obstacle detection with a focus on midair collision avoidance. Publications from the beginning until 2022 were searched in Scopus, IEEE, ACM, MDPI, and Web of Science databases. From the initial 647 publications obtained, 85 were finally selected and examined. The results show an increasing interest in this topic, especially in relation to object detection and tracking. Our study hypothesizes that the widespread access to commercial drones, the improvements in single-board computers, and their compatibility with computer vision libraries have contributed to the increase in the number of publications. The review also shows that the proposed algorithms are mainly tested using simulation software and flight simulators, and only 26 papers report testing with physical flying vehicles. This systematic review highlights other gaps to be addressed in future work. Several identified challenges are related to increasing the success rate of threat detection and testing solutions in complex scenarios.

## 1. Introduction

Although the sky may seem big enough for two flying vehicles to collide, the facts show that midair collisions still occur from time to time and are a major concern for aviation safety authorities. As a preventative measure, pilots are instructed to keep one eye out of the cockpit, scan the sky for potential threats, and be prepared to maneuver to avoid a potential accident [1,2]. However, this “see-and-avoid” rule has several important limitations. First, the pilot cannot look outside all the time, as he also has to check the instruments inside the cockpit, which measure, for example, air speed or engine temperature. The time spent looking inwards and outwards must therefore be perfectly balanced. Pilots who spend most of their time looking at the instruments, and this is especially true of novice pilots, endanger the aircraft by ignoring other elements circulating around them.

The “80–20” rule suggests that pilots should spend no less than 80% of their time looking out and no more than 20% of their time checking instruments. The 80 does not just refer to looking for other traffic, as the pilot also looks for visual cues used for navigation. In any case, even if a pilot or crew member could spend 100% of their time scanning the sky, this would not mean that no threat could escape the human eye. In fact, the fraction of our visual field that allows us to detect anything in the sky is extremely small. Therefore, for practical scanning, pilots are also instructed to follow a pattern, dividing the horizon into regions and taking a moment (1–2 s) to focus before moving on to the next region. Hence, the horizon is divided into nine regions; the pilot’s eye scans one ninth at a time. In other words, at least 89% of the horizon remains unattended at all times. This gives a clear idea of the chances of a threat escaping the human eye, especially when you consider that a light aircraft, such as a 9-meter-wingspan Piper PA-28 Cherokee, approaching head-on at 90 knots on a collision course, 5 seconds before impact, looks no bigger than a sparrow [3]. To make matters worse, the performance of the human eye can be reduced by cloud cover, glare from the sun, fatigue, and many other factors.

With today’s technologies, such as SSR (secondary surveillance radar), transponders, TCAS (traffic collision avoidance system) and, more recently, ADS-B (automatic dependent surveillance-broadcast), one might think that midair collisions should no longer occur. However, they do, because these technologies are not mandatory in all countries, airspaces, or aircraft. They are also fallible, because human factors still cause accidents. The new century has also brought a new player onto the scene. Since 2005, the use of unmanned aerial vehicles (UAVs) or drones in commercial applications has grown exponentially [4,5], increasing the need for safe path planning [6] and obstacle avoidance [7]. New commercial applications are not without risk, potentially causing damage and disrupting other aerial activities [8]. In 2017, a DJI Phantom 4 collided with a US Army UH-60M helicopter near Coney Island, New York. This is only the first documented case of a UAV collision with a manned aircraft, but the number of UAV sightings by pilots has increased dramatically in recent years. Aircraft collision avoidance is therefore a challenging problem due to the stochastic environment and uncertainty about the intentions of other aircraft [9].

For these reasons, and particularly in the case of manned and unmanned light aircraft in uncontrolled airspace and at aerodromes, various safety agencies and pilot associations are encouraging pilots and UAV users to install some form of electronic conspicuity (EC) device on their vehicles to be more aware of nearby aircraft [10,11,12]. An example of such EC technology is FLARM (FLight alARM, https://flarm.com/ accessed on 11 September 2023). The FLARM predicts potential conflicts and alerts the pilot with visual and audible warnings [13]. This device was originally designed for gliders, which are slower than light aircraft. The main limitation of these devices is compatibility, as a FLARM can only display air traffic that uses another matching FLARM. Incompatibility occurs, for example, when the communication solution is different due to the use of different frequencies (the US version of FLARM devices uses a different band than the European one) or different protocols (a FLARM device that has not been updated for a year is not compatible with the latest version of the protocol and will automatically shut down). In addition, some devices are active, i.e., they transmit and share their position with others, while others are passive, i.e., they listen to the transmissions of others but remain invisible to them (e.g., many portable air traffic detectors only listen to the transponders of other aircraft). In this “Tower of Babel” scenario, when communications fail or are absent, pilots should rely not solely on their eyes to detect threats, but on an artificial eye capable of scanning the sky faster, farther, wider, sharper, and more consistently.

The most used solution for preventing accidents is the use of radars. There are many types of radar used in aviation, but the most important is the primary surveillance radar (PSR). PSR detects the position and altitude of an aircraft by measuring the time it takes for radar waves to bounce off an aircraft and return to the radar antenna. These radar systems can detect both transponder-equipped and non-transponder-equipped aircraft. PSR is not perfect and has its limitations. PSR cannot identify the detected obstacle, and the required equipment is expensive [14]. Computer vision solutions have the advantage in this subject because the equipment is relatively cheap, and depending on the implementation, the solution can identify the incoming obstacle. Nevertheless, computer vision effectiveness can be impacted by light conditions. That is the reason why some researchers try to combine both approaches like [15] to overcome each approach’s limitations.

This systematic review, therefore, focuses on solutions that implement computer vision for obstacle avoidance in flight. Systematic reviews help researchers to learn about the current state of the art and to extend it with new studies. Computer vision is a combination of several algorithms that typically mimic human vision [16,17,18,19]. Inspired by the visual stimulus received by animals moving through the world, optical flow is defined as the pattern of apparent motion of objects, surfaces, and edges in a visual scene caused by the relative motion between an observer and a scene [20,21,22]. Optical flow can be applied to object segmentation, object detection, stereo disparity measurement, and motion compensation [23,24]. Another prominent approach to calculating apparent motion is the Kalman filter, an algorithm that helps to estimate unknown variables given observed measurements over time. The Kalman filter computes the motion vector of moving objects, making it possible to track them [25]. Because computer vision applications are relatively easy to implement, the number of publications on collision avoidance systems based on computer vision has increased greatly. Other computer vision algorithms for motion detection can be found in recent reviews [26,27].

Although several reviews have been carried out on the detection of moving objects using computer vision [28,29], to our knowledge, none has focused on the application of obstacle avoidance to light and unmanned aircraft. Furthermore, there are several reasons for undertaking the present systematic review. First, it was considered pertinent to conduct a comprehensive literature review on the detection of moving objects by computer vision on light aircraft, with the aim of providing an overview of existing solutions. Second, it was thought to identify possible gaps in existing studies on the detection of flying objects by computer vision in light and unmanned aircraft. Finally, it was relevant to identify topics for future studies related to the main topic of this systematic review.

This paper is organized as follows: The systematic review methodology, data extraction, research questions, search strategy, and answers to the research questions are described in Section 2. Section 3 contains the results and limitations. The discussion of the results is provided in Section 4. Finally, Section 5 presents the conclusions of the review.

## 2. Research Methodology

This systematic review was conducted using the common framework [30], and the reporting was done according to the PRISMA 2020 statement: an updated guideline for reporting systematic reviews [31].

### 2.1. Search Criteria

Five databases were searched for this systematic review: Scopus (Elsevier), IEEE Xplore (Institute of Electrical and Electronics Engineers), ACM Digital Library (Association for Computing Machinery), Multidisciplinary Digital Publishing Institute (MDPI), and WoS (Web of Science). The studies used in this systematic review were identified using the following search query: TITLE-ABS-KEY(((collision OR crash OR accident OR impact OR hit OR “air proximity” OR sense) AND (avoidance OR evasion OR prevention OR avoid)) AND (aircraft OR aerospace OR ((flying OR “unmanned aerial vehicle”)) AND (robot OR vehicle)) AND (vision OR visual) AND (detection OR tracking) AND (simulation OR simulator OR “virtual reality” OR “augmented reality”)).

Starting with the papers obtained through the search string described in the previous section, the following criteria were used to include the publications in our review:

- The papers used computer vision only to detect moving obstacles or threats.
- Object detection was used to avoid midair collisions in manned or unmanned aircraft.
- The papers were written in English.

Conversely, the following types of publications were excluded:

- Abstracts without full text.
- Systematic reviews, meta-analyses, and survey publications.

### 2.2. Search Process

**Figure 1:**
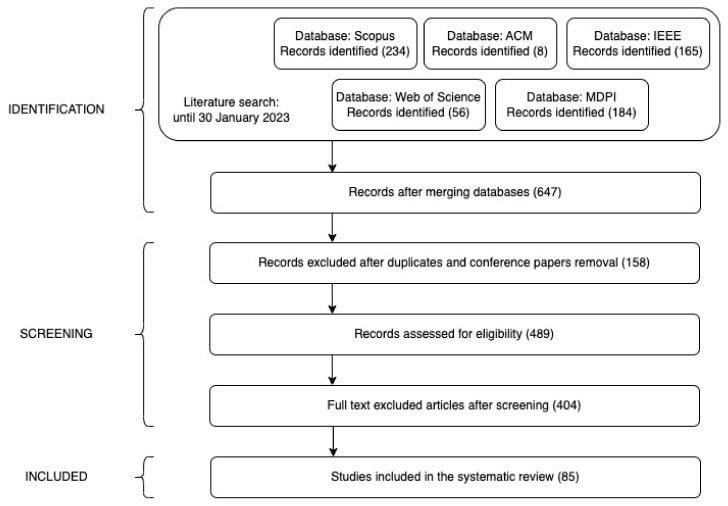
Search process.

The general description of the publication extraction and selection process is shown in Figure 1. The search string was queried in each of the five databases, yielding a total of 647 records. After removing 158 duplicate references and conference papers, one researcher independently filtered the remaining 489 articles by examining the full content of each publication. If the researcher was unsure whether to include or exclude an article, the publication was presented to the remaining researchers for discussion to reach a consensus decision. On completion of the screening process, 404 articles were excluded according to the remaining exclusion criteria, resulting in a final selection of 85 publications.

### 2.3. Research Directives

The six research questions (RQs) for this systematic review and their rationale were as follows:

RQ1. How many papers have been published on computer-vision-based moving obstacle detection for midair collision avoidance? Is there a time trend? 

Finding out how many articles have been published on the use of computer vision for a light aircraft to detect possible obstacles is intended to understand which technologies are most commonly used and to know the possible next research steps. It also seems appropriate to determine whether there is a time trend (increasing or decreasing) in the production of publications on this topic.

RQ2. How many cameras did the algorithms need? 

Investigating which type and number of cameras gives the best results for a particular algorithm or situation seems interesting for future studies. In general terms, a stereo camera has two or more image sensors to simulate human binocular vision, giving it the ability to perceive depth, unlike a monocular camera. Both stereo and monocular cameras are able to perform object detection, but only stereo cameras are able to calculate the distance to an object with high accuracy [32].

RQ3. What are the most commonly used computer vision techniques? 

The goal of vision recognition is to imitate or even surpass the human eye. Vision recognition processes are grouped as follows:

- Feature extraction is the identification of unique data in an image. Often lines and corners are good features because they provide large intensity contrasts. Feature extraction algorithms are the basis for object tracking and detection [33].
- Motion detection is the detection of changes in the physical position of the object. For static cameras, background subtraction algorithms can be used to detect motion. On the other hand, for moving cameras, optical flow can be used to detect the movement of pixels in the given image [34].
- Object detection is a set of computer vision tasks involving the identification of objects in images. This task requires a data set of labeled features to compare with an input image. Feature extraction algorithms are used to create the data sets [35].
- Object tracking. Given the initial state of a target object in one frame (position and size), object tracking estimates the states of the target object in subsequent frames. Tracking relies entirely on object detection. Tracking is much faster than detection because it already knows the appearance of the objects [36].
- Single-view geometry is the calculation of the geometry of an object using images from a single camera.

Visual recognition techniques are implemented using a variety of algorithms. The classification of published articles according to the visual recognition algorithms used provides an overview of the most commonly used algorithms in the literature and the situations in which they are most applicable.

RQ4. Which tools have been most commonly used to check the algorithms’ performance? 

The testing tools can be divided into those that have been carried out on the aircraft themselves, those that have been carried out using simulators, and a combination of the two. A flight simulator is a device that artificially recreates the flight of an aircraft and the environment in which it flies. It has the advantage that it can be used safely for pilot training, aircraft development, and the study of aircraft characteristics and controls. A simulator must be as close to reality as possible. It should therefore include the equations that govern the flight of the aircraft, its response to the application of flight controls, the effects of other aircraft systems, and the response of the aircraft to any external factors that threaten its flight. Obviously, different test tools may be used at each stage of an investigation into vision-based obstacle detection in flight. Testing in a controlled environment, such as a simulation, can help investigators identify problems with the algorithm in an early stage. A simulator can also prevent accidents caused by faulty algorithms. Once an algorithm has passed several tests in a controlled environment, the authors can start testing in the real world. Testing in an uncontrolled environment presents different challenges, such as wind, light sources, wind, or clouds. Therefore, a review of the testing methods used and their limitations is of interest for future studies.

RQ5. What kinds of flying vehicles were used to test the collision avoidance algorithms? 

The classification of the published papers according to the type of aircraft used to test their algorithms gives us an idea of the characteristics and limitations of the proposed solutions, and whether they are ready to be applied on a light or unmanned aircraft. Furthermore, the choice of UAVs for testing depends on many factors, such as resources, objectives, or legislation. Multirotor UAVs, airplane models, or helicopter models can be used for low-altitude test cases or environments with multiple obstacles, such as a city or a forest. On the other hand, aircraft can be used for test cases with high altitudes and speeds, taking into account that special permissions may be required.

RQ6. What kind of software has been used to perform computer simulations over the years? 

The classification of published articles according to the type of software used to perform their simulations indicates which type of software is trending or being abandoned. Different software may be used at different stages of the investigation, and some may be used as support. For testing the basic components of an algorithm in a numerical environment, Matlab and Simulink are probably a good choice. Flight simulators provide a more realistic environment in which investigators can infer the performance of the proposed solution. Finally, other software, such as the Robot Operating System (ROS) and Google Earth, could be used as supporting tools for the flight simulators.

## 3. Results

A final number of 85 publications were extracted using the process described above. References to the 85 papers are included in Appendix A. The number of resulting publications shows the growth and evolution of moving obstacle avoidance using computer vision from flying vehicles over the years. Here, we answer the six research questions presented above in relation to the selected bibliography.

RQ1. How many papers have been published on computer-vision-based moving obstacle detection for midair collision avoidance? Is there a time trend? 

**Figure 2:**
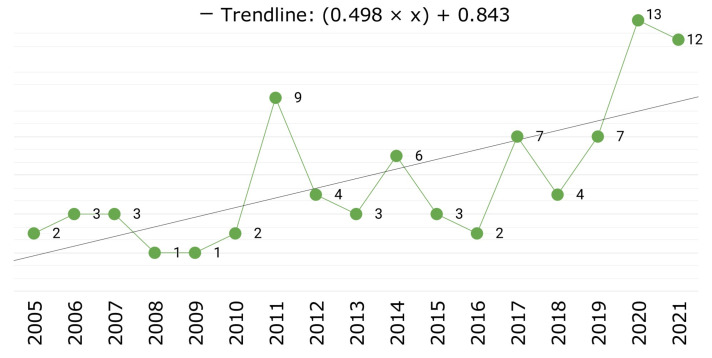
Publications over the years.

Figure 2 shows the number of articles published per year throughout the period studied. The period begins with only one publication found between 1964 and 1999; for this reason, it was excluded from the trend. This is followed by a 5-year period between 2000 and 2004 with no publications. Then there is a constant and regular number of publications of 2 or 3 articles per year from 2005 to 2010, with a drop to 1 publication in 2008 and 2009. The year 2011 is a turning point, where a rapid increase in published articles is observed, with 9 in that year, the third highest behind 12 in 2021. Recent years also show a significant interest in moving object avoidance based on computer vision, with 7 articles in 2019, 13 in 2020, and 12 in 2021. The year 2022 is a special case with only 2 articles, but the authors found several papers published in that year that focus on automatic flight navigation avoiding environmental obstacles. Overall, publications on this topic show an increasing trend line over the years.

RQ2. How many cameras did the algorithms need? 

**Figure 3:**
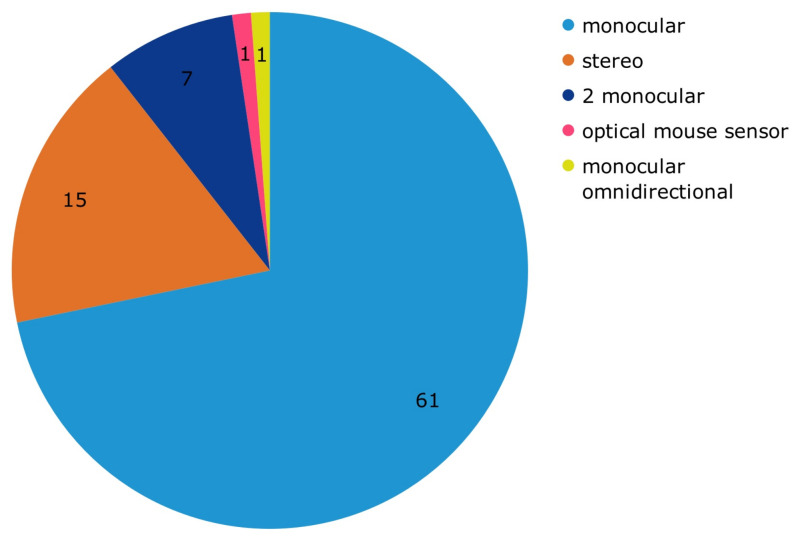
Categorization of papers by camera type.

The selected papers mainly distinguish between two types of cameras, stereoscopic and monocular. In addition, the works that use monocular cameras can be divided into four subgroups according to the number of monocular cameras used in the system: one, two, or even omnidirectional cameras, plus one studio that uses an optical mouse sensor ([P12]). As can be seen in Figure 3, 61 articles use a single monocular camera, followed by 15 publications using stereo cameras ([P3], [P10], [P27,] [P33], [P34], [P35], [P43], [P44], [P46], [P48], [P49], [P62], [P73], [P81], [P82]) and 7 papers using two monocular cameras ([P1], [P6], [P9], [P23], [P30], [P70], [P85]). Finally, we have 1 paper with an omnidirectional camera ([P42]). Thus, most of the papers use monocular cameras, which means that the authors focused on the detection of obstacles.

RQ3. What are the most commonly used computer vision techniques? 

**Figure 4:**
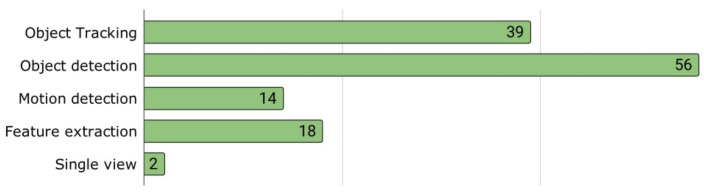
Number of papers using each vision recognition method.

**Figure 5:**
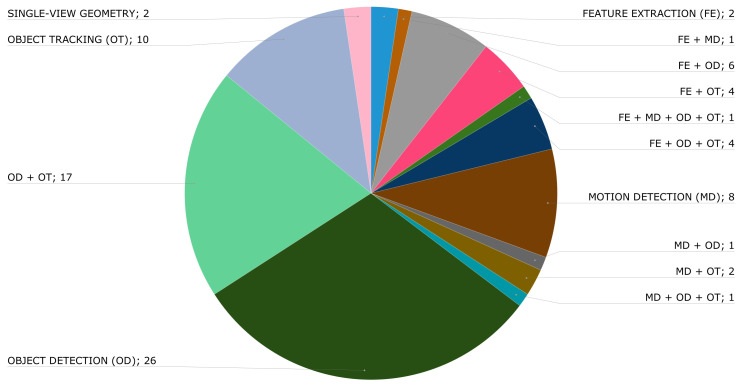
Combination of vision recognition processes in paper.

Vision recognition techniques are grouped into feature extraction, motion detection, object detection, object tracking, and single-view geometry. As can be seen in Figure 4, the most commonly used techniques are object detection (56 articles) and object tracking (39 articles). In this figure, some of the articles are counted more than once because the authors combine more than one technique in their solution. These combinations are shown in Figure 5.

In fact, the most common combination is object detection plus object tracking (OD + OT), with a total of 17 articles. Furthermore, each process can be implemented using different algorithms. Table 1 shows the algorithms used in each of them. According to our results, 65.88% of the selected publications used object detection alone or combined it with another computer vision algorithm, as shown in Table 2.

RQ4. Which tools have been most commonly used to check the algorithms’ performance? 

**Figure 6:**
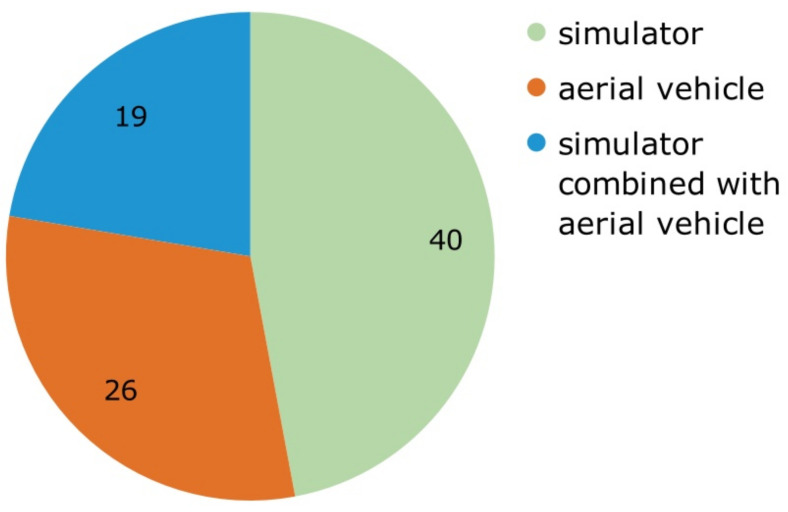
Categorization of papers by test method.

As can be seen in Figure 6, the papers used three different methods to test their findings: using only aerial vehicles (30.58%), using a combination of aerial vehicles and simulators (22.35%), and most papers using only simulators (47.05%). Testing with aerial vehicles is more expensive and can cause accidents. This may explain why most papers only use simulation to test their solutions.

RQ5. What kinds of flying vehicles were used to test the collision avoidance algorithms? 

**Figure 7:**
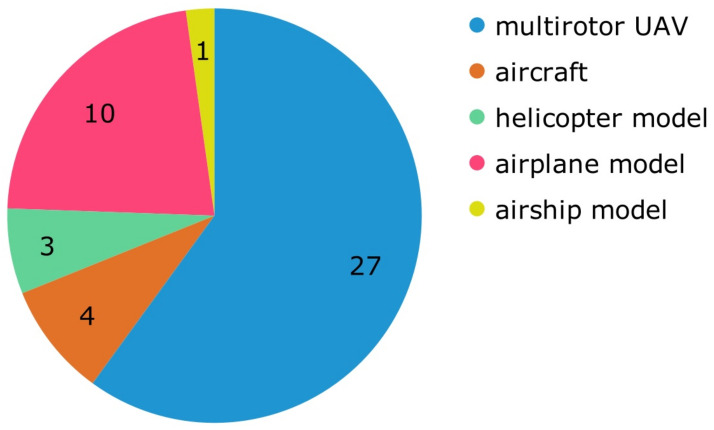
Categorization of papers using physical equipment by aerial vehicle.

**Figure 8:**
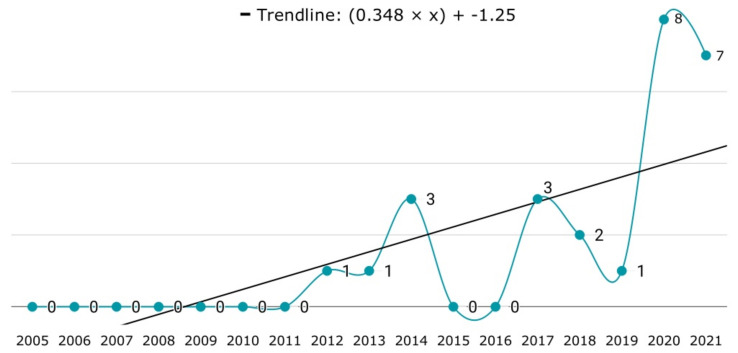
Use of multirotor UAVs over the years.

Almost half of the papers (47.05%) used some kind of simulation to test their algorithms. The rest of the papers used some kind of flying vehicle, as shown in Figure 7; they were divided into two groups. UAVs whose weight varies from 250 gm to 2 Kg and aircraft that need a pilot and can carry passengers. The UAVs group contains 27 publications that use multirotor UAVs. Some of the used UAVs are Parrot AR.Drone ([P23], [P55], [P76], [P77], [P79], [P82]), DJI Matrice 100 ([P48], [P80]), AscTec Pelican ([P34]), Spreading Wings S1000 ([P61]), and a custom drone ([P73], [P81]). Ten papers used airplane models ([P6], [P12], [P13], [P22], [P26], [P29], [P38], [P42], [P49], [P70]), 3 used helicopter models ([P1], [P3], [P39]), and 1 paper used an airship model ([P9]). For the second group, 4 papers used an aircraft ([P10], [P40], [P47], [P85]). The results indicate that the researchers prefer to use UAVs for testing their solutions. Figure 8 shows the use of multirotor UAVs over the years.

RQ6. What kind of software has been used to perform computer simulations over the years? 

**Figure 9:**
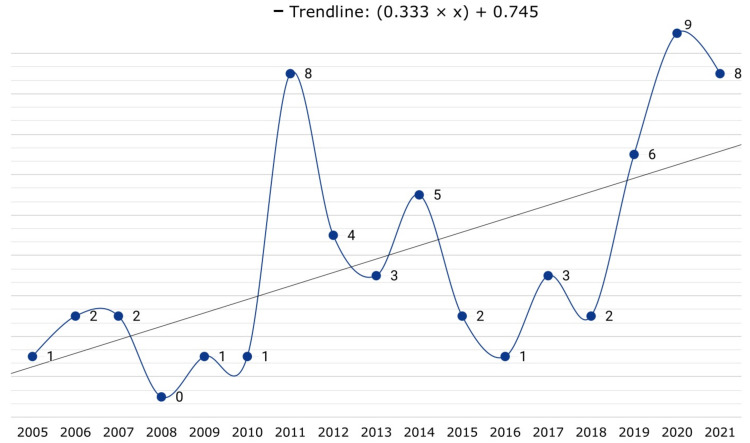
Use of simulation over the years.

The result of extracting the software used for simulations is shown in Table 3. Not all articles that carried out simulations mentioned the software used. Ten other articles describe developing their own software for the study. From 2010 to 2017, Matlab and Simulink were the preferred tools for simulation. However, from 2018 onwards, the preferences changed to specialized simulation software. The flight simulators found in the papers are FlightGear ([P15], [P21], [P25]), DJI flight simulator and Microsoft AirSim ([P48], [P83]), jMAVSim ([P52]), and X-Plane 11 ([P59]). As a special case, the paper [P69] used a 3D computer graphics software tool called Blender to create a simulation to test their solution. Figure 9 shows the number of publications using simulation over the years.

## 4. Discussion

To the best of our knowledge, this is the first systematic review of computer-vision-based moving obstacle detection for midair collision avoidance. The study included articles searched from inception to 2022, although only 23 years of publications were found (1999–2022). A total of 85 articles were selected from the 647 initial publications obtained from five databases (Scopus, IEEE, ACM, MDPI, and WoS). The results made it possible to evaluate the current situation of this growing field of knowledge.

The issue has attracted attention in recent years due to several factors. First, the availability of nonmilitary drones to the general public since 2006 [37,38] poses several challenges. The possibility of drones colliding not only with other unmanned aerial vehicles in the air but also with manned and passenger aircraft is a major concern today. In addition, the market offers a wide catalog of drones with distinctive features, such as integrated cameras, global positioning system devices, wireless connectivity, accelerometers, and altimeters. This easy access to drones also allows researchers to test different solutions without major risks [39].

### 4.1. Computer Vision

The second factor is computer vision. Computer vision began in the early 1970s as a way to mimic human vision, and it has continued to grow and improve ever since [40]. Thanks to many advances, computer vision libraries require less computing power to run their algorithms, making it more feasible for researchers to move their solutions to lower-end hardware [41].

Finally, single-board computers give researchers the ability to test on the fly without the need for heavy equipment [42]. A single-board computer is a single PC board with a processor, memory, and some form of I/O that allows it to function as a computer [43]. The size and weight of single-board computers make them perfect for mounting on a drone or light aircraft without affecting its performance in flight. In the mid-1970s, the “dyna-micro” was one of the first true single-board computers. The next major development in single-board computers came in 2008 from BeagleBoard.org, which created a low-cost, open-source development board called the BeagleBoard [43].

According to the reviewed papers, the detection of moving obstacles with computer vision starts with the images provided by a camera or a group of cameras. Computer vision cannot be accurate without obtaining good images with the best possible resolution. In the included studies, the majority of publications used a single monocular camera (71.76%). Papers using stereo cameras represent 17.64% of the publications. This is probably due to the fact that applications using stereo cameras are computationally expensive compared with those using monocular cameras [44].

The captured images must then be processed using computer algorithms to detect possible obstacles. The most commonly used vision recognition process identified in the papers was object detection. Object detection involves identifying a specific object in successive images. The perfect object detection algorithm must be fast and accurate. Object detection can be complemented by object tracking, which uses information from the previous frame to track objects and is faster than object detection [45].

Grouping the selected papers by the method used to test the collision avoidance algorithm shows that almost half of the publications (47.05%) use only computer simulations to verify and validate their solutions. Using these simulations is cheaper and safer than using a manned or unmanned aircraft. It is safer because it avoids the risks of testing a collision avoidance algorithm in real flight, where accidents can occur and have costly—or even fatal—consequences, especially in the early stages of development when the solution is less mature and more prone to error.

### 4.2. Testing Tools

Researchers prefer a controlled environment to carry out tests and improve their solutions before real-world trials take place. Matlab is a programming and numerical computing platform. Matlab was released to the public in 1984. Matlab can be used for data analysis, data visualization, algorithm development, and more [46]. Matlab’s ease of use and large database of built-in algorithms make it one of the preferred methods for testing algorithms. However, since 2018, authors prefer to use flight simulators (5 papers from 2018 to 2021), Gazebo (4 papers from 2019 (Acropolis) to 2021 (Fortress)), and Blender v2.91 (1 paper in 2020). New improvements and increased realism in off-the-shelf flight simulators may be the reason for authors to switch to this software, in particular FlightGear v2020.3.18 and X-Plane v12.0. FlightGear is a free, open-source, multiplatform flight simulator. The first version was released in 1997. FlightGear was used in two papers described at RQ6, immediately after the launch of a major release, FlightGear 2.0, in 2010. X-Plane is a flight simulator available since 1995. In 2017, the engine received a major update (v11.0), providing greater graphical realism [47,48]. The recent release of the popular Microsoft Flight Simulator (v1.31.22.0), after many years since the last update, may make it another option to consider in future releases.

The use of real drones as a testing method for validating collision avoidance algorithms began in 2012, as shown in Figure 8. The use of drones helps researchers create a more realistic yet controlled testing environment, reducing interference when assessing the effectiveness of an algorithm. Although the Federal Aviation Administration issued a commercial drone permit in 2006, drones were not widely available at the time [49]. It was not until 2010 that the company Parrot released Parrot AR.Drone 1.0 [50]. This drone was a huge commercial success, selling over half a million units. Parrot AR.Drone 2.0 was released in 2012 [51]. In 2013, the Chinese company DJI launched the first camera-equipped drone called Phantom 1 [52]. In 2018, the same company launched the DJI Mavic Air. This drone was designed to be portable, powerful, and accessible to enthusiasts of all levels. More interestingly, the DJI Mavic Air added an obstacle avoidance system for safety in the air [53]. Unfortunately, the authors could not find any details on the obstacle avoidance system used by DJI.

It is noteworthy that only four publications reported the use of a manned aircraft in the tests. As discussed above, the use of simulators does not incur the cost of using real vehicles and reduces the hazards. Small quadrotor UAVs are also affordable and have a very low cost compared with manned aircraft. However, it should be noted that the solutions tested on quadrotor UAVs may not be directly applicable to light-manned aircraft or even larger drones due to factors such as vehicle speed, flight altitude, and weather conditions, to name a few.

### 4.3. Obstacles and Future Work

The algorithms, solutions, and proposals described in the articles included in this systematic review are not yet free from shortcomings that should be addressed in the next revisions, which represents an opportunity for future work and developments in this field. Some problems are related to errors, inaccuracies, and false positives ([P7], [P24], [P41], [P42]). For example, [P42] reports 90% hits in near scenarios but 40% false alarms in far ones, which also shows the importance of testing in different scenarios to realize the limitations of a proposal. Indeed, many authors are willing to test their solutions in additional scenarios, especially in more complex, crowded, and noisy environments, as the next step of their research ([P4], [P7], [P15], [P25], [P31], [P39], [P59], [P61]). Simulation software plays an important role here. For example, [P48] uses the minimalist proprietary DJI flight simulator, but the authors claim that another, more appropriate simulation software would be needed to further improve their algorithms. A ground-based video dataset, such as the one presented in [P32], may be the solution for evaluating video-based detection and avoidance systems.

However, beyond more realistic simulations and video datasets, many authors would like to extend their test to the real world, i.e., to perform real flight tests with their solution embedded in a real manned or unmanned aircraft ([P4], [P8], [P18], [P27], [P48], [P59], [P60]). Real tests reveal other problems related to vehicle speed ([P11], [P30], [P61]) or energy consumption ([P48]). For example, in [P11], a single 2D camera is used to generate a 3D model of the scene, but the lower is the speed, the less information is obtained. On the contrary, in [P30], the authors point out that the faster the drone moves, the more blurred the video becomes, which reduces the accuracy of the algorithm.

The limited field of view of the cameras is another problem ([P25]). Some authors propose to address this in future work using additional cameras ([P61]) or other sensors, such as a depth sensor ([P60]), laser rangefinder ([P11]), or global positioning system (GPS) ([P18]). For example, in [P18], the authors plan to use GPS and stereo vision to determine the positions of both the vehicle and the obstacles in real tests. The tracking of obstacles and their direction and speed is another problem to be solved ([P8], [P10], [P18], [P25], [P30], [P41]). In particular, distance estimation is a real challenge ([P41]). The correct spatial positioning of an obstacle is key for avoidance algorithms ([P8], [P27], [P33], [P41], [P54]), where further research is needed to improve maneuvering, minimize effort, and reduce deviation after avoidance. Finally, one paper proposed to investigate collision avoidance approaches for multiple intruders ([P39]).

For manned aircraft, one problem with detecting and avoiding obstacles in the air is how to warn pilots of a potential threat. Again, preparing test scenarios with manned aircraft is expensive, and we believe that technologies such as virtual reality and augmented reality would help. Such technologies have grown considerably in recent years [54,55]. For example, immersive virtual reality could be combined with flight simulator software to reproduce test scenarios for prototyping and testing warning solutions. Augmented reality could be used to simulate approaching threats in flight and could also lead to new ways of alerting and guiding pilots to avoid a collision. It is worth noting that these technologies were included as keywords in the search, but no matching results were found. We believe that they are promising technologies that should be explored in future approaches.

From the discussions so far, it can be concluded that the publications are still at an early stage of research and that further progress is needed to find solutions to the problems identified.

## 5. Conclusions

See and avoid is a basic procedure that pilots must learn and apply during flight. Various technologies have been introduced to avoid midair collisions, but accidents still occur because they are neither mandatory in all airspaces nor suitable for all aircraft. Technology can also fail and human error can occur, as was sadly demonstrated in 1986 when a Piper Cherokee light aircraft collided with the vertical stabilizer of a DC-9 airliner over Los Angeles International Airport (California) because the Piper did not have a Mode C transponder, which would have alerted others of its presence. In addition, neither pilot appeared to have seen the other aircraft. Computer vision can assist pilots in this task, leading to solutions that match or exceed the performance of the human eye in this task, complementing the pilot’s functions, or being used in autonomous systems to avoid obstacles and other flying vehicles.

This systematic review has shown that there is a continuing interest in research into computer-vision-based moving obstacle detection for midair collision avoidance. A total of 85 papers were analyzed. The results show that researchers’ attention is mostly focused on motion and object detection, as well as object tracking. In addition, the preferred way to test solutions is to use simulation software, such as Matlab in early papers or flight simulators in recent papers. Only a few papers have reported testing with physical flying vehicles, namely, quadrotor UAVs, aircraft and helicopter models, and only one manned aircraft. Some of the current shortcomings, and therefore future challenges, are related to increasing the success rate of detection and, in particular, testing solutions in many different, more complex, and noisy scenarios. The use of real vehicles in real scenarios is considered by most authors to be an outstanding task.

In terms of possible improvements, the articles reviewed point to improving the detection of moving obstacles, with a focus on avoiding target loss due to occlusion. Multiple obstacle avoidance is also an important feature that seems to deserve more attention. Testing in new environments will improve the effectiveness of the algorithms, such as obstacle detection at night, which will likely require the use of night vision cameras. The authors agree that the algorithms need to extract more information about the obstacle, such as direction, speed, and distance. There is growing support for the use of stereoscopic cameras to improve the calculation of distance. It is also worth mentioning that it can be very helpful to know the current position of the aircraft via GPS. As for simulators, there is a desire for more accurate simulated environments. A good simulator can help scientists extensively test their solutions in a variety of situations where an incident in a real environment could be dangerous or costly.

Looking beyond the single UAV or manned aircraft, networking technologies in the Internet of Things (IoT) era could help share obstacle data detected by one or more vehicles, adding information such as distance or bearing that could be helpful in congested areas, and even acting as a multicamera distributed obstacle detection system in a group of vehicles, such as UAV hives. Integrating computer-vision-based algorithms with current electronic conspicuity (EC) devices, which already use their technologies to broadcast their own position to other compatible devices within range, would facilitate this.

Finally, it is essential to note that for manned aircraft, none of the selected publications investigated how to alert the pilot or the person controlling the UAV that an incoming obstacle has been detected. Future publications should investigate more effective user interfaces for alerting a pilot of incoming obstacles. Virtual and augmented reality are technologies that would play an essential role in this regard [56].

## Figures and Tables

**Table 1:** Vision recognition algorithms.

Method	Algorithms	Paper
Feature extraction	Speeded up robust feature (SURF)	[P5], [P79]
	Sobel, Prewitt, Roberts edge detection	[P10]
	Threshold, blurring, Canny edge detection	[P30]
	Good features to track	[P32]
	Canny edge detection, Shi-Tomasi feature detector	[P38]
	Grayscale, Canny edge detection	[P41]
	SIFT, SURF, homography	[P47]
	Harris corner detection	[P57]
	ORB	[P60]
	Shi-Tomasi corner detection	[P63]
	Canny edge detection	[P66], [P70]
	Difference of Gaussians	[P67], [P76]
	Morphological processing, Sobel edge detection	[P68]
	ResNet-50 CNN	[P78]
	Convolutional neural network (CNN)	[P81]
Motion detection	Optical flow and scene reconstruction	[P2], [P14]
	Optical flow and inertial data	[P7]
	Optical flow	[P12], [P18], [P24], [P36], [P59], [P61], [P70], [P85]
	Background subtraction	[P13]
	Grayscale and binary foveal processors	[P42]
	Feature reprojection and matching	[P60]
Object detection	Disparity map	[P3], [P48]
	LGMD-based neural network	[P9]
	Extended and unscented Kalman filters	[P15]
	Hidden Markov model (HMM)	[P16]
	Shi-Tomasi corner detector	[P20]
	Edge detection, color segmentation	[P21]
	CMO combined HMM, CMO combined Viterbi-based filtering	[P22]
	Camshift algorithm	[P23]
	Single-point feature	[P6], [P29]
	Depth map	[P27]
	Hough transform and contour detection	[P30]
	Unscented Kalman filter	[P33]
	Disparity space	[P34], [P35]
	Viola–Jones algorithm, morphological detection algorithm	[P37]
	Erosion and dilation morphological operators	[P38]
	CMO combined HMM	[P39]
	CMO, bottom hat filtering, top hat filtering, standard deviation	[P40]
	Contour detection	[P41], [P54]
	Grayscale and binary foveal processors	[P42]
	Haar cascade	[P43]
	Triangulation, depth map	[P44]
	CMO, bottom hat filtering, adaptive contour-based morphology,	
	Viterbi-based filtering, HMM	[P45]
	Epipolar geometry	[P46]
	Background subtraction	[P47]
	Stereo block matching	[P49]
	CNN with SegNet architecture	[P51], [P55]
	Single-shot detector SSD	[P52]
	MobileNet-SSD CNN	[P56]
	YOLOv2	[P58], [P78]
	Semiglobal matching (SGM), DBSCAN	[P62]
	Lucas–Kanade optical flow	[P63]
	ConvLSTM network	[P64]
	Bottom hat filtering, HMM	[P65]
	U-Net CNN	[P67]
	MSER blob detector	[P68]
	YOLOv3	[P69], [P70], [P71], [P72], [P73], [P75], [P80], [P82]
	Horn–Schunck optical flow	[P74]
	Custom artificial neural network	[P76]
	Gaussian filter, Farnebäck optical flow	[P77]
	Recursive neural network (RNN)	[P81]
	Pix2Pix (optical flow)	[P83]
	YOLOv4	[P84]
	Dynamic object contour extraction	[P85]
Object tracking	Extended Kalman filter	[P1], [P4], [P5], [P8], [P11]
	Kalman filter	[P3], [P42], [P44], [P53], [P58], [P70], [P72], [P75], [P78], [P79]
	Imagination-augmented agents (I2A)	[P6]
	Three nested Kalman filters	[P7]
	SIFT, Kalman filter	[P13]
	Hidden Markov model	[P16]
	Lucas–Kanade optical flow	[P20], [P28], [P32]
	Camshift algorithm	[P23]
	Extended and unscented Kalman filters	[P25]
	Single-point feature	[P26]
	Visual predictive control	[P29]
	Unscented Kalman filter	[P31], [P33]
	Kanade–Lucas–Tomasi	[P37]
	Lucas–Kanade optical tracker	[P38]
	Close-minus-open and hidden Markov model	[P39]
	Template matching, Kalman filtering	[P40]
	Distant-based and distance-agnostic	[P41]
	Camshift	[P43]
	HMM, ad hoc Viterbi temporal filtering	[P47]
	Parallel tracking and mapping, extended Kalman filter	[P50]
	MAVSDK (collision avoidance)	[P52]
	Kanade–Lucas–Tomasi (KLT)	[P57]
	SORT (Kalman filter, Hungarian algorithm)	[P73]
Single-view geometry	Single-view geometry and closest point of approach	[P17]
	Visual servoing and camera geometry	[P19]

**Table 2:** Articles related to computer vision methods.

Year	Feature Extraction	Single-View Geometry	Object Detection	Motion Detection	Object Tracking
1999	–	–	–	–	[P1]
2005	–	–	[P3]	[P2]	[P3]
2006	[P5]	–	–	–	[P4], [P5], [P6]
2007	–	–	[P9]	[P7]	[P7], [P8]
2008	[P10]	–	–	–	–
2009	–	–	–	–	[P11]
2010	–	[P17], [P19]	–	[P12], [P13]	[P13]
2011	–	–	[P15], [P16], [P20], [P21], [P22]	[P14], [P18]	[P16], [P20]
2012	–	–	[P23], [P26]	[P24]	[P23], [P25], [P26]
2013	–	–	[P27], [P29]	–	[P28], [P29]
2014	[P30], [P32]	–	[P30], [P33], [P34], [P35]	–	[P31], [P32], [P33]
2015	[P38]	–	[P37], [P38]	[P36]	[P37], [P38]
2016	–	–	[P39], [P40]	–	[P39], [P40]
2017	[P41], [P47]	–	[P41], [P42], [P43], [P44], [P45], [P46], [P47]	[P42]	[P41], [P42], [P43], [P44], [P47]
2018	–	–	[P48], [P49], [P51]	–	[P50]
2019	[P57]	–	[P52], [P54], [P55], [P56], [P58]	–	[P52], [P53], [P57], [P58]
2020	[P60], [P63], [P66], [P67], [P68], [P81]	–	[P62], [P63], [P64], [P65], [P67], [P68], [P69], [P80], [P81]	[P59], [P60], [P61]	–
2021	[P70], [P76], [P78]	–	[P70], [P71], [P72], [P73], [P74], [P75], [P76], [P77], [P78], [P82], [P84], [P85]	[P70], [P85]	[P70], [P72], [P73], [P75], [P78]
2022	[P79]	–	[P83]	–	[P79]

**Table 3:** Software used to run simulations (some publications use more than one).

Year	Flight Simulator	Gazebo	Matlab	Simulink	Robot Operating System (ROS)	Google Earth	Blender
2010	–	–	[P13]	–	–	–	–
2011	[P15], [P16]	–	[P15], [P18], [P21]	[P15]	–	–	–
2012	[P25]	[P23]	[P24], [P25], [P26]	[P24], [P25]	[P23]	[P24]	–
2013	–	–	[P29]	–	–	–	–
2014	–	–	[P31]	–	–	–	–
2015	–	–	[P36]	[P36]	–	–	–
2017	–	–	[P46]	[P46]	–	–	–
2018	[P48]	–	–	–	–	–	–
2019	[P52], [P57]	[P56]	–	–	–	–	–
2020	[P59]	[P60]	[P68]	–	–	–	[P69]
2021	[P71]	[P72], [P73]	[P70], [P74]	–	[P82]	–	–
2022	[P83]	–	–	–	–	–	–

## Data Availability

The data presented in this study are available on request from the corresponding author.

## Abbreviations

The following abbreviations are used in this manuscript:

ADS-B	automatic dependent surveillance-broadcast
FLARM	FLight alARM
ROS	Robot Operating System
SSR	secondary surveillance radar
TCAS	traffic collision avoidance system
UAV	unmanned aerial vehicle

## Appendix A

The list of the 85 publications resulted after the publication extraction and selection process.

[P1] S. Fürst, E-D. Dickmanns, “A vision based navigation system for autonomous aircraft,” *Robotics and Autonomous Systems*, vol. 28, no. 2–3, pp. 173–184, 1999.
[P2] A. Roderick, J. Kehoe, R. Lind, “Vision-Based Navigation Using Multi-Rate Feedback from Optic Flow and Scene Reconstruction,” *AIAA Guidance, Navigation, and Control Conference and Exhibit*, 2005.
[P3] R-K. Mehra, J. Byrne, J. Boskovic, “Flight testing of a fault-tolerant control and vision-based obstacle avoidance system for uavs,” *In Proceedings of the 2005 Association for Unmanned Vehicle Systems International (AUVSI) Conference, North America*, 2005.
[P4] Y. Watanabe, A. Calise, E. Johnson, J. Evers, “Minimum-Effort Guidance for Vision-Based Collision Avoidance,” *AIAA Atmospheric Flight Mechanics Conference and Exhibit*, 2006.
[P5] R. Prazenica, A. Kurdila, R. Sharpley, J. Evers, “Vision-based geometry estimation and receding horizon path planning for UAVs operating in urban environments,” *2006 American Control Conference*, 2006.
[P6] J-C. Zufferey, D. Floreano, “Fly-inspired visual steering of an ultralight indoor aircraft,” *IEEE Transactions on Robotics*, vol. 22, no. 1, pp. 137–146, 2006.
[P7] F. Kendoul, I. Fantoni, G. Dherbomez, “Three Nested Kalman Filters-Based Algorithm for Real-Time Estimation of Optical Flow, UAV Motion and Obstacles Detection,” *Proceedings 2007 IEEE International Conference on Robotics and Automation*, 2007.
[P8] Y. Watanabe, A. Calise, E. Johnson, “Vision-Based Obstacle Avoidance for UAVs,” *AIAA Guidance, Navigation and Control Conference and Exhibit*, 2007.
[P9] S. Bermudez i Badia, P. Pyk, P-F-M-J. Verschure, “A fly-locust based neuronal control system applied to an unmanned aerial vehicle: the invertebrate neuronal principles for course stabilization, altitude control and collision avoidance,” *The International Journal of Robotics Research*, vol. 26, no. 7, pp. 759–772, 2007.
[P10] E. Hanna, P. Straznicky, R. Goubran, “Obstacle Detection for Low Flying Unmanned Aerial Vehicles Using Stereoscopic Imaging,” *2008 IEEE Instrumentation and Measurement Technology Conference*, 2008.
[P11] S. Shah, E. Johnson, “3-D Obstacle Detection Using a Single Camera,” *AIAA Guidance, Navigation, and Control Conference*, 2009.
[P12] J-C. Zufferey, A. Beyeler, D. Floreano, “Autonomous flight at low altitude with vision-based collision avoidance and GPS-based path following,” *2010 IEEE International Conference on Robotics and Automation*, 2010.
[P13] L. Mejias, S. McNamara, J. Lai, J. Ford, “Vision-based detection and tracking of aerial targets for UAV collision avoidance,” *2010 IEEE/RSJ International Conference on Intelligent Robots and Systems*, 2010.
[P14] A-R. Arvai, J-J. Kehoe, R. Lind, “Vision-based navigation using multi-rate feedback from optic flow and scene reconstruction,” *The Aeronautical Journal*, vol. 115, no. 1169, pp. 411–420, 2011.
[P15] B. Vanek, T. Peni, J. Bokor, T. Zsedrovits, A. Zarandy, T. Roska, “Performance analysis of a vision only Sense and Avoid system for small UAVs,” *AIAA Guidance, Navigation, and Control Conference*, 2011.
[P16] A. Wainwright, J. Ford, J. Lai, “Fat and thin adaptive HMM filters for vision based detection of moving targets,” *In Proceedings of the 2011 Australasian Conference on Robotics and Automation (ACRA 2011)*, pp. 1–10, 2011.
[P17] H. Choi, Y. Kim, I. Hwang, “Vision-Based Reactive Collision Avoidance Algorithm for Unmanned Aerial Vehicle,” *AIAA Guidance, Navigation, and Control Conference*, 2011.
[P18] D-W. Yoo, D-Y. Won, M-J. Tahk, “Optical Flow Based Collision Avoidance of Multi-Rotor UAVs in Urban Environments,” *International Journal of Aeronautical and Space Sciences*, vol. 12, no. 3, pp. 252–259, 2011.
[P19] D. Lee, H. Lim, H-J. Kim, “Obstacle avoidance using image-based visual servoing integrated with nonlinear model predictive control,” *IEEE Conference on Decision and Control and European Control Conference*, 2011.
[P20] Y. Chen, A. Abushakra, J. Lee, “Vision-Based Horizon Detection and Target Tracking for UAVs,” *Advances in Visual Computing*, pp. 310–319, 2011.
[P21] T. Zsedrovits, A. Zarandy, B. Vanek, T. Peni, J. Bokor, T. Roska, “Collision avoidance for UAV using visual detection,” *2011 IEEE International Symposium of Circuits and Systems (ISCAS)*, 2011.
[P22] J. Lai, L. Mejias, J-J. Ford, “Airborne vision-based collision-detection system,” *Journal of Field Robotics*, vol. 28, no. 2, pp. 137–157, 2011.
[P23] M-A. Olivares-Mendez, P. Campoy, I. Mellado-Bataller, L. Mejias, “See-and-avoid quadcopter using fuzzy control optimized by cross-entropy,” *2012 IEEE International Conference on Fuzzy Systems*, 2012.
[P24] A. Eresen, N. İmamoğlu, M. Önder Efe, “Autonomous quadrotor flight with vision-based obstacle avoidance in virtual environment,” *Expert Systems with Applications*, vol. 39, no. 1, pp. 894–905, 2012.
[P25] B. Vanek, T. Péni, Zarándy, J. Bokor, T. Zsedrovits, T. Roska, “Performance Characteristics of a Complete Vision Only Sense and Avoid System,” *AIAA Guidance, Navigation, and Control Conference*, 2012.
[P26] A. Mcfadyen, P. Corke, L. Mejias, “Rotorcraft collision avoidance using spherical image-based visual servoing and single point features,” *2012 IEEE/RSJ International Conference on Intelligent Robots and Systems*, 2012.
[P27] J. Park, Y. Kim, “Obstacle Detection and Collision Avoidance of Quadrotor UAV Using Depth Map of Stereo Vision,” *AIAA Guidance, Navigation, and Control (GNC) Conference*, 2013.
[P28] C-M. Huang, M-L. Chiang, L-C. Fu, “Adaptive Visual Servoing of Micro Aerial Vehicle with Switched System Model for Obstacle Avoidance,” *2013 IEEE International Conference on Systems, Man, and Cybernetics*, 2013.
[P29] A. Mcfadyen, L. Mejias, P. Corke, C. Pradalier, “Aircraft collision avoidance using spherical visual predictive control and single point features,” *2013 IEEE/RSJ International Conference on Intelligent Robots and Systems*, 2013.
[P30] L-K. Kong, J. Sheng, A. Teredesai, “Basic Micro-Aerial Vehicles (MAVs) obstacles avoidance using monocular computer vision,” *2014 13th International Conference on Control Automation Robotics & Vision (ICARCV)*, 2014.
[P31] B. Vanek, T. Peni, P. Bauer, J. Bokor, “Vision only sense and avoid: A probabilistic approach,” *2014 American Control Conference*, 2014.
[P32] A. Carrio, C. Fu, J. Pestana, P. Campoy, “A ground-truth video dataset for the development and evaluation of vision-based Sense-and-Avoid systems,” *2014 International Conference on Unmanned Aircraft Systems (ICUAS)*, 2014.
[P33] A-K. Tripathi, R-G. Raja, R. Padhi, “Reactive Collision Avoidance of UAVs with Stereovision Camera Sensors using UKF,” *IFAC Proceedings Volumes*, vol. 47, no. 1, pp. 1119–1125, 2014.
[P34] R. Brockers, Y. Kuwata, S. Weiss, L. Matthies, “Micro air vehicle autonomous obstacle avoidance from stereo-vision,” *SPIE Proceedings*, 2014.
[P35] L. Matthies, R. Brockers, Y. Kuwata, S. Weiss, “Stereo vision-based obstacle avoidance for micro air vehicles using disparity space,” *2014 IEEE International Conference on Robotics and Automation (ICRA)*, 2014.
[P36] P. Agrawal, A. Ratnoo, D. Ghose, “Vision Based Obstacle Detection and Avoidance for UAVs Using Image Segmentation,” *AIAA Guidance, Navigation, and Control Conference*, 2015.
[P37] M. Clark, Z. Kern, R-J. Prazenica, “A Vision-Based Proportional Navigation Guidance Law for UAS Sense and Avoid,” *AIAA Guidance, Navigation, and Control Conference*, 2015.
[P38] S. Huh, S. Cho, Y. Jung, D-H. Shim, “Vision-based sense-and-avoid framework for unmanned aerial vehicles,” *IEEE Transactions on Aerospace and Electronic Systems*, vol. 51, no. 4, pp. 3427–3439, 2015.
[P39] Y. Lyu, Q. Pan, C. Zhao, Y. Zhang, J. Hu, “Vision-based UAV collision avoidance with 2D dynamic safety envelope,” *IEEE Aerospace and Electronic Systems Magazine*, vol. 31, no. 7, pp. 16–26, 2016.
[P40] G. Fasano, D. Accardo, A-E. Tirri, A. Moccia, E-D. Lellis, “Sky Region Obstacle Detection and Tracking for Vision-Based UAS Sense and Avoid,” *Journal of Intelligent & Robotic Systems*, vol. 84, no. 1–4, pp. 121–144, 2015.
[P41] A. Morgan, Z. Jones, R. Chapman, S. Biaz, “An unmanned aircraft "see and avoid" algorithm development platform using opengl and opencv,” *Journal of Computing Sciences in Colleges*, vol. 33, no. 2, pp. 229–236, 2017.
[P42] P. Bauer, A. Hiba, J. Bokor, “Monocular image-based intruder direction estimation at closest point of approach,” *2017 International Conference on Unmanned Aircraft Systems (ICUAS)*, 2017.
[P43] H. Sedaghat-Pisheh, A-R. Rivera, S. Biaz, R. Chapman, R., “Collision avoidance algorithms for unmanned aerial vehicles using computer vision,” *Journal of Computing Sciences in Colleges*, vol. 33, no. 2, pp. 191–197. 2017.
[P44] Y. Lyu, Q. Pan, C. Zhao, J. Hu, “Autonomous Stereo Vision Based Collision Avoid System for Small UAV,” *AIAA Information Systems-AIAA Infotech @ Aerospace*, 2017.
[P45] D. Bratanov, L. Mejias, J-J. Ford, “A vision-based sense-and-avoid system tested on a ScanEagle UAV,” *2017 International Conference on Unmanned Aircraft Systems (ICUAS)*, 2017.
[P46] A. Ramani, H-E. Sevil, A. Dogan, “Determining intruder aircraft position using series of stereoscopic 2-D images,” *2017 International Conference on Unmanned Aircraft Systems (ICUAS)*, 2017.
[P47] T-L. Molloy, J-J. Ford, L. Mejias, “Detection of aircraft below the horizon for vision-based detect and avoid in unmanned aircraft systems,” *Journal of Field Robotics*, vol. 34, no. 7, pp. 1378–1391, 2017.
[P48] B. Ruf, S. Monka, M. Kollmann, M. Grinberg, “Real-Time On-Board Obstacle Avoidance for Uavs Based on Embedded Stereo Vision,” *The International Archives of the Photogrammetry, Remote Sensing and Spatial Information Sciences*, vol. XLII-1, pp. 363-370, 2018.
[P49] A-J. Barry, P-R. Florence, R. Tedrake, “High-speed autonomous obstacle avoidance with pushbroom stereo,” *Journal of Field Robotics*, vol. 35, no. 1, pp. 52–68, 2017.
[P50] D. Mercado, P. Castillo, R. Lozano, “Sliding mode collision-free navigation for quadrotors using monocular vision,” *Robotica*, vol. 36, no. 10, pp. 1493–1509, 2018.
[P51] J. James, J-J. Ford, T-L. Molloy, “Learning to Detect Aircraft for Long-Range Vision-Based Sense-and-Avoid Systems,” *IEEE Robotics and Automation Letters*, vol. 3, no. 4, pp. 4383–4390, 2018.
[P52] V. Varatharasan, A-S-S. Rao, E. Toutounji, J-H. Hong, H-S. Shin, “Target Detection, Tracking and Avoidance System for Low-cost UAVs using AI-Based Approaches,” *2019 Workshop on Research, Education and Development of Unmanned Aerial Systems (RED UAS)*, 2019.
[P53] Y. Zhang, W. Wang, P. Huang, Z. Jiang, “Monocular Vision-Based Sense and Avoid of UAV Using Nonlinear Model Predictive Control,” *Robotica*, vol. 37, no. 9, pp. 1582–1594, 2019.
[P54] C. Li, X. Xie, F. Luo, “Obstacle Detection and Path Planning Based on Monocular Vision for Unmanned Aerial Vehicles,” *2019 Chinese Automation Congress (CAC)*, 2019.
[P55] J. James, J-J. Ford, T-L. Molloy, “Below Horizon Aircraft Detection Using Deep Learning for Vision-Based Sense and Avoid,” *2019 International Conference on Unmanned Aircraft Systems (ICUAS)*, 2019.
[P56] D-S. Levkovits-Scherer, I. Cruz-Vega, J. Martinez-Carranza, “Real-Time Monocular Vision-Based UAV Obstacle Detection and Collision Avoidance in GPS-Denied Outdoor Environments Using CNN MobileNet-SSD,” *Advances in Soft Computing*, pp. 613–621, 2019.
[P57] D. Zuehlke, N. Prabhakar, M. Clark, T. Henderson, R-J. Prazenica, “Vision-Based Object Detection and Proportional Navigation for UAS Collision Avoidance,” *AIAA Scitech 2019 Forum*, 2019.
[P58] R. Opromolla, G. Fasano, D. Accardo, “Experimental assessment of vision-based sensing for small UAS sense and avoid,” *2019 IEEE/AIAA 38th Digital Avionics Systems Conference (DASC)*, 2019.
[P59] P. Rzucidło, T. Rogalski, G. Jaromi, D. Kordos, P. Szczerba, A. Paw, “Simulation studies of a vision intruder detection system,” *Aircraft Engineering and Aerospace Technology*, vol. 92, no. 4, pp. 621–631, 2020.
[P60] F. Gomes, T. Hormigo, R. Ventura, “Vision based real-time obstacle avoidance for drones using a time-to-collision estimation approach,” *2020 IEEE International Symposium on Safety, Security, and Rescue Robotics (SSRR)*, 2020.
[P61] N. Urieva, J. McDonald, T. Uryeva, A-S. Rose Ramos, S. Bhandari, “Collision Detection and Avoidance using Optical Flow for Multicopter UAVs,” *2020 International Conference on Unmanned Aircraft Systems (ICUAS)*, 2020.
[P62] J. Park, N. Cho, S. Lee, “Reactive Collision Avoidance Algorithm for UAV Using Bounding Tube against Multiple Moving Obstacles,” *IEEE Access*, vol. 8, pp. 218131–218144, 2020.
[P63] W-K. Ang, W-S. Teo, O. Yakimenko, “Enabling an EO-Sensor-Based Capability to Detect and Track Multiple Moving Threats Onboard sUAS Operating in Cluttered Environments,” *Proceedings of the 2019 2nd International Conference on Control and Robot Technology*, 2019.
[P64] S. Hussaini, J. Martin, J. Ford, “Vision based aircraft detection using deep learning with synthetic data,” *In Proceedings of the Australasian Conference on Robotics and Automation (ACRA)*, 2020.
[P65] J. James, J-J. Ford, T-L. Molloy, “A Novel Technique for Rejecting Non-Aircraft Artefacts in Above Horizon Vision-Based Aircraft Detection,” *2020 International Conference on Unmanned Aircraft Systems (ICUAS)*, 2020.
[P66] R. Raheem Nhair, T-A. Al-Assadi, “Vision-Based Obstacle Avoidance for Small Drone Using Monocular Camera,” *IOP Conference Series: Materials Science and Engineering*, vol. 928, no. 3, pp. 032048, 2020.
[P67] M. Petho, A. Nagy, T. Zsedrovits, “A bio-motivated vision system and artificial neural network for autonomous UAV obstacle avoidance,” *2020 3rd International Seminar on Research of Information Technology and Intelligent Systems (ISRITI)*, 2020.
[P68] T-K. Hao, O. Yakimenko, “Assessment of an Effective Range of Detecting Intruder Aerial Drone Using Onboard EO-Sensor,” *2020 6th International Conference on Control, Automation and Robotics (ICCAR)*, 2020.
[P69] Y-C. Lai, Z-Y. Huang, “Detection of a Moving UAV Based on Deep Learning-Based Distance Estimation,” *Remote Sensing*, vol. 12, no. 18, pp. 3035, 2020.
[P70] C. Kang, H. Chaudhry, C-A. Woolsey, K. Kochersberger, “Development of a Peripheral–Central Vision System for Small Unmanned Aircraft Tracking,” *Journal of Aerospace Information Systems*, vol. 18, no. 9, pp. 645–658, 2021.
[P71] E. Cetin, C. Barrado, E. Pastor, “Counter a Drone and the Performance Analysis of Deep Reinforcement Learning Method and Human Pilot,” *2021 IEEE/AIAA 40th Digital Avionics Systems Conference (DASC)*, 2021.
[P72] S. Karlsson, C. Kanellakis, S-S. Mansouri, G. Nikolakopoulos, “Monocular Vision-Based Obstacle Avoidance Scheme for Micro Aerial Vehicle Navigation,” *2021 International Conference on Unmanned Aircraft Systems (ICUAS)*, 2021.
[P73] G. Chen, W. Dong, X. Sheng, X. Zhu, H. Ding, “An Active Sense and Avoid System for Flying Robots in Dynamic Environments,” *IEEE/ASME Transactions on Mechatronics*, vol. 26, no. 2, pp. 668–678, 2021.
[P74] K-S. Karreddula, A-K. Deb, “Center of View Based Guidance Angles for Collision-Free Autonomous Flight of UAV,” *2021 International Symposium of Asian Control Association on Intelligent Robotics and Industrial Automation (IRIA)*, 2021.
[P75] W-L. Leong, P. Wang, S. Huang, Z. Ma, H. Yang, J. Sun, Y. Zhou, M-R. Abdul Hamid, S. Srigrarom, R. Teo, “Vision-Based Sense and Avoid with Monocular Vision and Real-Time Object Detection for UAVs,” *2021 International Conference on Unmanned Aircraft Systems (ICUAS)*, 2021.
[P76] M. Petho, T. Zsedrovits, “UAV obstacle detection with bio-motivated computer vision,” *2021 17th International Workshop on Cellular Nanoscale Networks and their Applications (CNNA)*, 2021.
[P77] H-Y. Lin, X-Z. Peng, “Autonomous Quadrotor Navigation with Vision Based Obstacle Avoidance and Path Planning,” *IEEE Access*, vol. 9, pp. 102450–102459, 2021.
[P78] R. Opromolla, G. Fasano, “Visual-based obstacle detection and tracking, and conflict detection for small UAS sense and avoid,” *Aerospace Science and Technology*, vol. 119, pp. 107167, 2021.
[P79] R-P. Padhy, P-K. Sa, F. Narducci, C. Bisogni, S. Bakshi, “Monocular Vision Aided Depth Measurement from RGB Images for Autonomous UAV Navigation,” *ACM Transactions on Multimedia Computing, Communications, and Applications*, 2022.
[P80] T. Zhang, X. Hu, J. Xiao, G. Zhang, “A Machine Learning Method for Vision-Based Unmanned Aerial Vehicle Systems to Understand Unknown Environments,” *Sensors*, vol. 20, no. 11, pp. 3245, 2020.
[P81] D. Pedro, J-P. Matos-Carvalho, F. Azevedo, R. Sacoto-Martins, L. Bernardo, L. Campos, J-M. Fonseca, A. Mora, “FFAU-Framework for Fully Autonomous UAVs,” *Remote Sensing*, vol. 12, no. 21, pp. 3533, 2020.
[P82] L-O. Rojas-Perez, J. Martinez-Carranza, “Towards Autonomous Drone Racing without GPU Using an OAK-D Smart Camera,” *Sensors*, vol. 21, no. 22, pp. 7436, 2021.
[P83] T. Shimada, H. Nishikawa, X. Kong, H. Tomiyama, “Pix2Pix-Based Monocular Depth Estimation for Drones with Optical Flow on AirSim,” *Sensors*, vol. 22, no. 6, pp. 2097, 2022.
[P84] H. Alqaysi, I. Fedorov, F-Z. Qureshi, M. O’Nils, “A Temporal Boosted YOLO-Based Model for Birds Detection around Wind Farms,” *Journal of Imaging*, vol. 7, no. 11, pp. 227, 2021.
[P85] P. Rzucidło, G. Jaromi, T. Kapuściński, D. Kordos, T. Rogalski, P. Szczerba, “In-Flight Tests of Intruder Detection Vision System,” *Sensors*, vol. 21, no. 21, pp. 7360, 2021.

## References

[1] Federal Aviation Administration (2021). How to Avoid a Mid Air Collision—P-8740-51. https://www.faasafety.gov/gslac/ALC/libview_normal.aspx?id=6851.

[2] Federal Aviation Administration (2016). Airplane Flying Handbook, FAA-H-8083-3B.

[3] UK Airprox Board (2017). When every second counts. Airprox Saf. Mag..

[4] Akbari Y., Almaadeed N., Al-maadeed S., Elharrouss O. (2021). Applications, databases and open computer vision research from drone videos and images: A survey. Artif. Intell. Rev..

[5] Yang X., Wei P. (2021). Autonomous Free Flight Operations in Urban Air Mobility with Computational Guidance and Collision Avoidance. IEEE Trans. Intell. Transp. Syst..

[6] Jiang Y., Wu Q., Zhang G., Zhu S., Xing W. (2021). A diversified group teaching optimization algorithm with segment-based fitness strategy for unmanned aerial vehicle route planning. Expert Syst. Appl..

[7] Shin S.Y., Kang Y.W., Kim Y.G. (2020). Reward-driven U-Net training for obstacle avoidance drone. Expert Syst. Appl..

[8] Ghasri M., Maghrebi M. (2021). Factors affecting unmanned aerial vehicles’ safety: A post-occurrence exploratory data analysis of drones’ accidents and incidents in Australia. Saf. Sci..

[9] Bertram J., Wei P., Zambreno J. (2022). A Fast Markov Decision Process-Based Algorithm for Collision Avoidance in Urban Air Mobility. IEEE Trans. Intell. Transp. Syst..

[10] Srivastava A., Prakash J. (2023). Internet of Low-Altitude UAVs (IoLoUA): A methodical modeling on integration of Internet of “Things” with “UAV” possibilities and tests. Artif. Intell. Rev..

[11] Jenie Y.I., van Kampen E.J., Ellerbroek J., Hoekstra J.M. (2018). Safety Assessment of a UAV CD&R System in High Density Airspace Using Monte Carlo Simulations. IEEE Trans. Intell. Transp. Syst..

[12] Uzochukwu S. (2019). I can see clearly now. Microlight Fly. Mag..

[13] Šimák V., Škultéty F. (2020). Real time light-sport aircraft tracking using SRD860 band. Transp. Res. Procedia.

[14] Vabre P. (2009). Air Traffic Services Surveillance Systems, Including an Explanation of Primary and Secondary Radar. Victoria, Australia: The Airways Museum & Civil Aviation Historical Society. http://www.airwaysmuseum.comSurveillance.htm.

[15] Vitiello F., Causa F., Opromolla R., Fasano G. (2022). Detection and tracking of non-cooperative flying obstacles using low SWaP radar and optical sensors: An experimental analysis. Proceedings of the 2022 International Conference on Unmanned Aircraft Systems (ICUAS).

[16] Huang T. Computer vision: Evolution and promise. Proceedings of the 1996 CERN School of Computing.

[17] Belmonte L.M., Morales R., Fernández-Caballero A. (2019). Computer vision in autonomous unmanned aerial vehicles—A systematic mapping study. Appl. Sci..

[18] Górriz J.M., Ramírez J., Ortíz A., Martínez-Murcia F.J., Segovia F., Suckling J., Leming M., Zhang Y.D., Álvarez Sánchez J.R., Bologna G. (2020). Artificial intelligence within the interplay between natural and artificial computation: Advances in data science, trends and applications. Neurocomputing.

[19] Ángel Madridano A., Al-Kaff A., Martín D., de la Escalera A. (2021). Trajectory planning for multi-robot systems: Methods and applications. Expert Syst. Appl..

[20] Horn B.K., Schunck B.G. (1981). Determining optical flow. Artif. Intell..

[21] Delgado A.E., López M.T., Fernández-Caballero A. (2010). Real-time motion detection by lateral inhibition in accumulative computation. Eng. Appl. Artif. Intell..

[22] López-Valles J.M., Fernández M.A., Fernández-Caballero A. (2007). Stereovision depth analysis by two-dimensional motion charge memories. Pattern Recognit. Lett..

[23] Liu S. (2009). Object Trajectory Estimation Using Optical Flow. Master’s Thesis.

[24] Almansa-Valverde S., Castillo J.C., Fernández-Caballero A. (2012). Mobile robot map building from time-of-flight camera. Expert Syst. Appl..

[25] Chen S.Y. (2012). Kalman Filter for Robot Vision: A Survey. IEEE Trans. Ind. Electron..

[26] Tang J., Duan H., Lao S. (2023). Swarm intelligence algorithms for multiple unmanned aerial vehicles collaboration: A comprehensive review. Artif. Intell. Rev..

[27] Al-Kaff A., Martín D., García F., de la Escalera A., María Armingol J. (2018). Survey of computer vision algorithms and applications for unmanned aerial vehicles. Expert Syst. Appl..

[28] Cebollada S., Payá L., Flores M., Peidró A., Reinoso O. (2021). A state-of-the-art review on mobile robotics tasks using artificial intelligence and visual data. Expert Syst. Appl..

[29] Llamazares A., Molinos E.J., Ocaña M. (2020). Detection and Tracking of Moving Obstacles (DATMO): A Review. Robotica.

[30] Arksey H., O’Malley L. (2005). Scoping studies: Towards a methodological framework. Int. J. Soc. Res. Methodol..

[31] Page M.J., McKenzie J.E., Bossuyt P.M., Boutron I., Hoffmann T.C., Mulrow C.D., Shamseer L., Tetzlaff J.M., Akl E.A., Brennan S.E. (2021). The PRISMA 2020 statement: An updated guideline for reporting systematic reviews. BMJ.

[32] Stanoev A., Audinet N., Tancock S., Dahnoun N. Real-time stereo vision for collision detection on autonomous UAVs. Proceedings of the 2017 IEEE International Conference on Imaging Systems and Techniques (IST).

[33] Jiang X. Feature extraction for image recognition and computer vision. Proceedings of the 2009 2nd IEEE International Conference on Computer Science and Information Technology.

[34] Manchanda S., Sharma S. Analysis of computer vision based techniques for motion detection. Proceedings of the 2016 6th International Conference-Cloud System and Big Data Engineering (Confluence).

[35] Redmon J., Divvala S., Girshick R., Farhadi A. You Only Look Once: Unified, Real-Time Object Detection. Proceedings of the 2016 IEEE Conference on Computer Vision and Pattern Recognition (CVPR).

[36] Wu Y., Lim J., Yang M.H. Online Object Tracking: A Benchmark. Proceedings of the 2013 IEEE Conference on Computer Vision and Pattern Recognition.

[37] Kellermann R., Biehle T., Fischer L. (2020). Drones for parcel and passenger transportation: A literature review. Transp. Res. Interdiscip. Perspect..

[38] Kindervater K.H. (2016). The emergence of lethal surveillance: Watching and killing in the history of drone technology. Secur. Dialogue.

[39] Hussein M., Nouacer R., Corradi F., Ouhammou Y., Villar E., Tieri C., Castiñeira R. (2021). Key technologies for safe and autonomous drones. Microprocess. Microsyst..

[40] Ortmeyer C. (2011). Computer Vision: Algorithms and Applications.

[41] Feng X., Jiang Y., Yang X., Du M., Li X. (2019). Computer vision algorithms and hardware implementations: A survey. Integration.

[42] Chamola V., Kotesh P., Agarwal A., Gupta N., Guizani M. (2021). A Comprehensive Review of Unmanned Aerial Vehicle Attacks and Neutralization Techniques. Ad Hoc Netw..

[43] Kindervater K.H. (2014). Then and now: A brief history of single board computers. Electron. Des. Uncovered.

[44] Fernández-Caballero A., López M.T., Saiz-Valverde S. (2008). Dynamic stereoscopic selective visual attention (DSSVA): Integrating motion and shape with depth in video segmentation. Expert Syst. Appl..

[45] Joshi P., Escrivá D., Godoy V. (2016). OpenCV by Example: Enhance Your Understanding of Computer Vision and Image Processing by Developing Real-World Projects in OpenCV 3.

[46] Moler C., Little J. (2020). A History of MATLAB. Proc. ACM Program. Lang..

[47] Yu L., He G., Zhao S., Wang X., Shen L. (2020). Design and implementation of a hardware-in-the-loop simulation system for a tilt trirotor UAV. J. Adv. Transp..

[48] Kumar A., Yoon S., Kumar V.R.S. (2020). Mixed reality simulation of high-endurance unmanned aerial vehicle with dual-head electromagnetic propulsion devices for earth and other planetary explorations. Appl. Sci..

[49] Dronethusiast (2019). The History of Drones (Drone History Timeline from 1849 to 2019). https://www.dronethusiast.com/history-of-drones/.

[50] Dormehl L. (2018). The History of Drones in 10 Milestones. https://www.digitaltrends.com/cool-tech/history-of-drones/.

[51] Pollicino J. (2012). Parrot Unveils AR.Drone 2.0 with 720p HD Camera, Autonomous Video-Recording, We Go Hands-On. https://www.engadget.com/2012-01-08-parrot-unveils-ar-drone-2-0-with-720p-hd-camera-autonomous-vide.html.

[52] DJI (2021). Phantom. https://www.dji.com/es/phantom.

[53] DrDrone.ca (2018). Timeline of DJI Drones: From the Phantom 1 to the Mavic Air. https://www.drdrone.ca/blogs/drone-news-drone-help-blog/timeline-of-dji-drones.

[54] Grand View Research (2021). Augmented Reality Market Size, Share & Trends Analysis Report By Component, By Display (HMD & Smart Glass, HUD, Handheld Devices), By Application, By Region, And Segment Forecasts, 2021–2028. https://www.grandviewresearch.com/industry-analysis/augmented-reality-market.

[55] Grand View Research (2021). Virtual Reality Market Size, Share & Trends Analysis Report by Technology (Semi & Fully Immersive, Non-immersive), By Device (HMD, GTD), by Component (Hardware, Software), by Application, and Segment Forecasts, 2021–2028. https://www.grandviewresearch.com/industry-analysis/virtual-reality-vr-market.

[56] Bustamante A., Belmonte L.M., Morales R., Pereira A., Fernández-Caballero A. (2022). Video Processing from a Virtual Unmanned Aerial Vehicle: Comparing Two Approaches to Using OpenCV in Unity. Appl. Sci..

